# Genome-wide Analysis of the Alternative Splicing Profiles Revealed Novel Prognostic Index for Kidney Renal Cell Clear Cell Carcinoma

**DOI:** 10.7150/jca.36998

**Published:** 2020-01-14

**Authors:** Li Gao, Rong-quan He, Zhi-guang Huang, Yi-wu Dang, Yong-yao Gu, Hai-biao Yan, Sheng-hua Li, Gang Chen

**Affiliations:** 1 Department of Pathology, First Affiliated Hospital of Guangxi Medical University, Nanning, Guangxi Zhuang Autonomous Region 530021, P. R. China; 2 Department of Medical Oncology, First Affiliated Hospital of Guangxi Medical University, Nanning, Guangxi Zhuang Autonomous Region 530021, P. R. China; 3 Department of Urology, First Affiliated Hospital of Guangxi Medical University, Nanning, Guangxi Zhuang Autonomous Region 530021, P. R. China

**Keywords:** alternative splicing, prognostic index, splicing factor, kidney clear renal cell carcinoma, the cancer genome atlas.

## Abstract

Alternative splicing (AS) is a major mechanism that greatly enhanced the diversity of proteome. Mounting evidence demonstrated that aberration of AS are important steps for the initiation and progression of human cancers. Here, we comprehensively investigated the association between whole landscape of AS profiles and the survival outcome of renal cell carcinoma (RCC) patients using RNA-seq data from TCGA SpliceSeq. Because of the limited number size of deaths in kidney chromophobe renal cell carcinoma (KICH) and papillary renal cell carcinoma (KIRP) TCGA cohorts, we only conducted survival analysis in kidney clear renal cell carcinoma (KIRC). We further constructed prognostic index (PI) based on prognosis-related AS events and built correlation network for splicing factors and prognosis-related AS events. According to the results, a total of 5351 AS events in 3522 genes were significantly correlated with the overall survival (OS) of kidney clear cell renal cell carcinoma (KIRC) patients. Seven of the PI models exhibited preferable prognosis-predicting capacity for KIRC with PI-ALL reaching the highest area under curve value of 0.875. The splicing regulatory network between splicing factors and prognosis-related AS events depicted a tangled web of relationships between them. One of the splicing factors: KHDRBS3 was validated by immunohistochemistry to be down-regulated in KIRC tissues. In conclusion, the powerful efficiency of risk stratification of PI models indicated the potential of AS signature as promising prognostic markers for KIRC and the splicing regulation network provided possible genetic mechanism of KIRC.

## Introduction

Renal cell carcinoma (RCC) is the most prevalent type of adult kidney cancer and is responsible for 14970 new deaths in 2018 worldwide 1, 2. RCC can be histological divided into three major subtypes with different genetic drivers and clinical courses: clear cell renal cell carcinoma (ccRCC), papillary renal cell carcinoma (PRCC) (including type1 and type2) and chromophobe renal cell carcinoma (ChRCC) 3. Although good effects have been achieved in the treatment of localized RCC using nephrectomy, advanced RCC remained incurable with poor survival outcome due to resistance to radiotherapy and chemotherapy targeting tumor vasculature 4-7. Therefore, it is crucial to find new prognostic predictors for the guidance of clinical therapy of RCC.

Alternative splicing (AS) is a pivotal process that increases the protein complexity through enabling the generation of multiple splice isoforms from an individual gene 8, 9, during which specific introns were selectively excised from the pre-mRNA and residual exons were rejoined 10. A growing body of researches suggested that aberration of AS are important steps for the initiation and progression of human cancers 11-13. In RCC, disturbance of AS of genes such as EZH2, PKM and FGFR2 have been observed by previous studies and isoform switches of these genes played active roles in the proliferation, growth and invasion of RCC 8, 14, 15, which demonstrated the immense value of AS in developing novel anti-cancer strategies for RCC. However, the prognostic significance of AS events was investigated for single or few genes in the past research and rarely has researchers worked out AS and splicing factor-based risk signature from the prognostic evaluation of whole AS or splicing factor profiles in RCC. The regulation mechanism of AS and splicing factor in KIRC has not been fully elucidated.

The feasibility of AS-based risk signature have been proved in non-small cell lung cancer, ovarian cancer, bladder cancer and gastrointestinal pan-adenocarcinomas 16-19, where AS-based risk signature have excellent distinguishing ability for the prognosis of low-risk and high-risk patients. Thus, we attempted to offer novel approach to prognostic stratification for RCC patients from the respective of AS and splicing factor. In this study, the most shining highlight of the work lies in that we for the first time conducted comprehensive assessment of the prognostic value of whole landscape of AS events in three major subtypes of RCC with available data from the cancer genome atlas (TCGA) and further constructed prediction models based on prognostic AS events and splicing factors for kidney renal clear cell carcinoma (KIRC). We also endeavored to comprehend the interaction mechanism between AS and splicing factor events on prognosis of KIRC patients through constructing regulation network of AS and splicing factors.

## Results

### Prognosis-related AS Events from univariate Cox regression analysis

**Figure 1:**
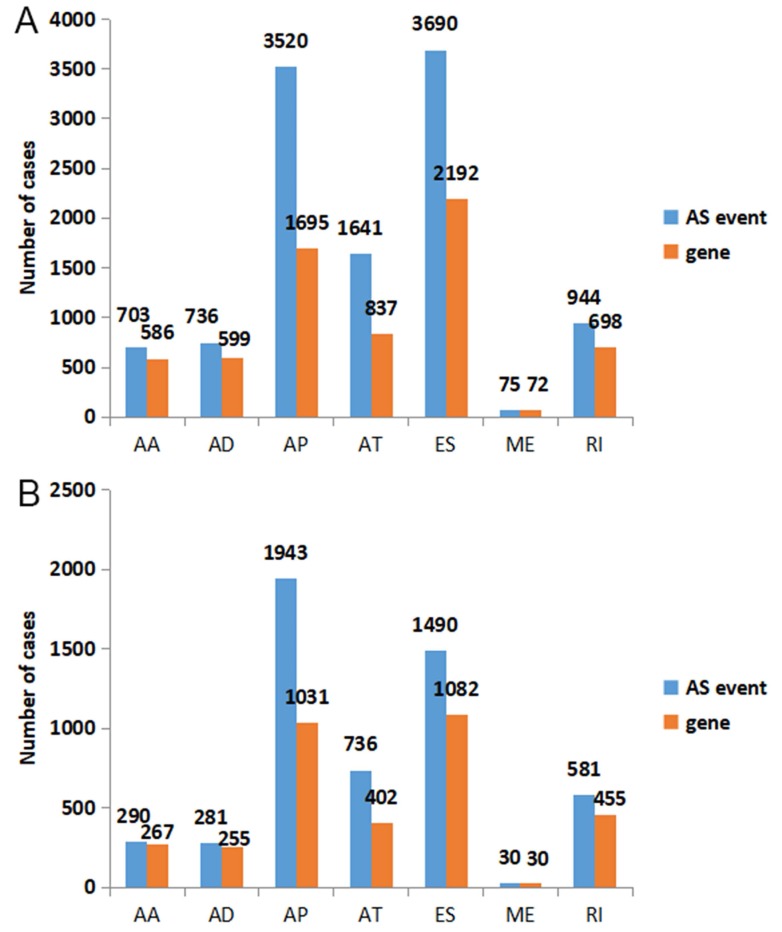
The distribution of all AS events and prognosis-related AS events in KIRC. A: Blue bars and orange bars indicated the number of AS events and the corresponding genes in each splicing type, respectively. B: The distribution of prognosis-related AS events in KIRC. Blue bars and orange bars indicated the number of prognosis-related AS events and the corresponding genes in each splicing type, respectively. AA: alternate acceptor site; AD: alternate donor site; AP: alternate promoter; AT: alternate terminator; ES: exon skip; ME: mutually exclusive exons; RI: retained intron.

**Figure 2:**
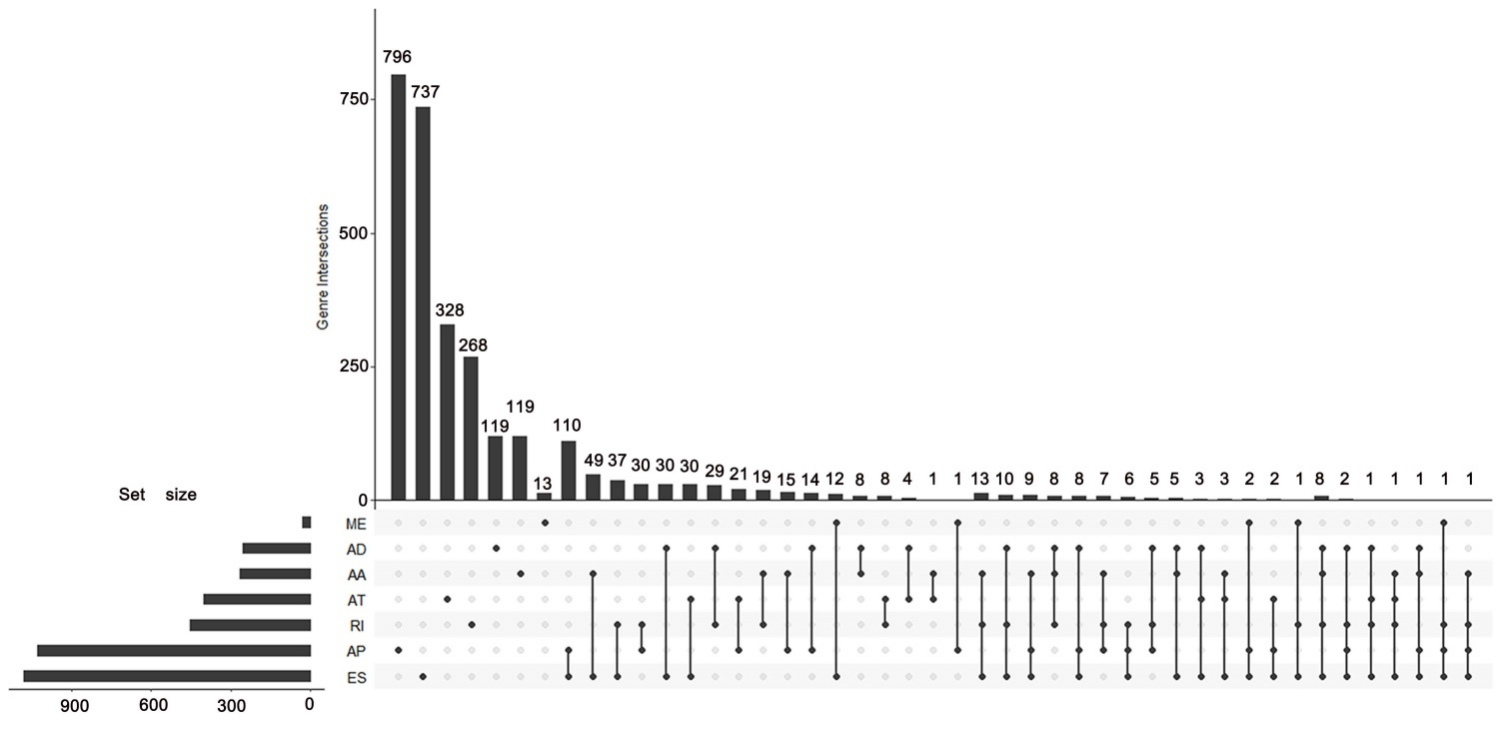
UpSet plot of survival-associated AS events. The UpSet plot illustrates the interactions among seven types of survival-associated alternative splicing (AS) events in KIRC. A single gene could have up to four types of prognosis-related AS events.

According to the including criteria of eligible cases, a total of 62 KICH, 468 KIRC, 122 KIRP type 1 and 53 KIRP type 2 patients were enrolled in this study. The distribution of AS events in the above major subtypes of RCC was summarized in Figure 1A. For all major subtypes of RCC, exon skip (ES) was the predominant splicing type with the highest number of AS events. Because of the limited number size of deaths in KICH and KIRP TCGA cohorts, we only conducted survival analysis in KIRC. Detailed clinical information for the included 468 KIRC patients was summarized in Table 1. Results from univariate Cox regression analysis revealed thousands of prognosis-related AS events in KIRC, consisting of 290 alternate acceptor site (AAs) in 267 genes, 281 alternate donor site (ADs) in 255 genes, 1943 alternate promoter (AP)s in 1031 genes, 736 alternate terminator (AT)s in 402 genes, 1490 ESs in 1082 genes, 30 mutually exclusive exons (MEs) in 30 genes and 581 retained intron (RIs) in 455 genes (Figure 1B). As the upset plot illustrated, a single gene might have up to four prognosis-related AS events (Figure 2).

### Protein-to-protein interaction (PPI) network and pathway annotation for genes of prognosis-related AS events

**Figure 3:**
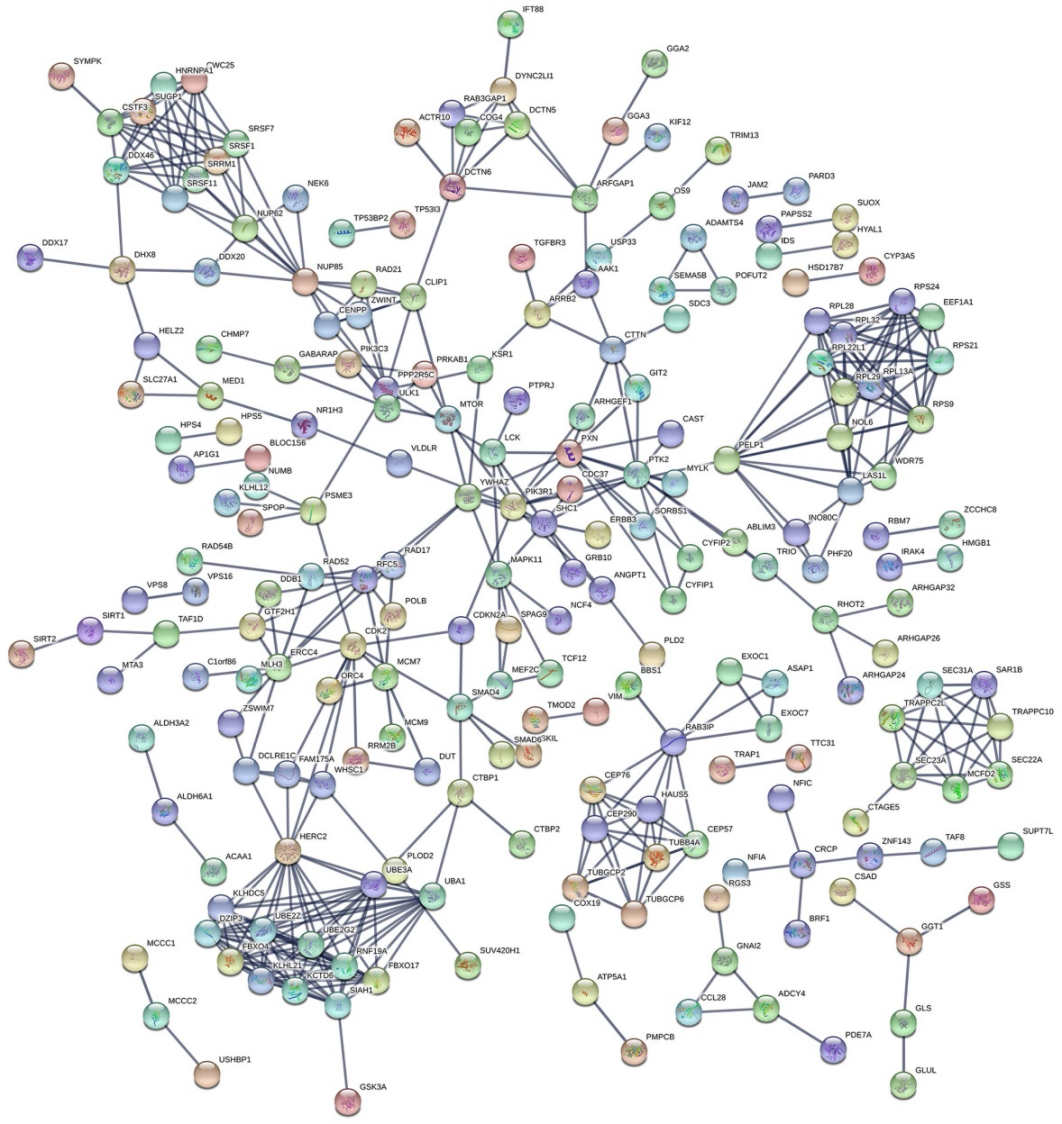
PPI network for genes of top 1000 prognosis-related AS events. The PPI network, comprised of 220 nodes and 466 links, described the interactions between genes corresponding to top 1000 prognosis-related AS events. Nodes with different colors represented different proteins and the edges between nodes represented known interactions or predicted interactions between these proteins.

The PPI network in Figure 3 displayed the interactions between genes of top 1000 prognosis-related AS events in KIRC. Calculation of connection degrees returned the following list of hub genes: HERC2, UBE3A, UBA1, SIAH1 and RPS9.

**Figure 4:**
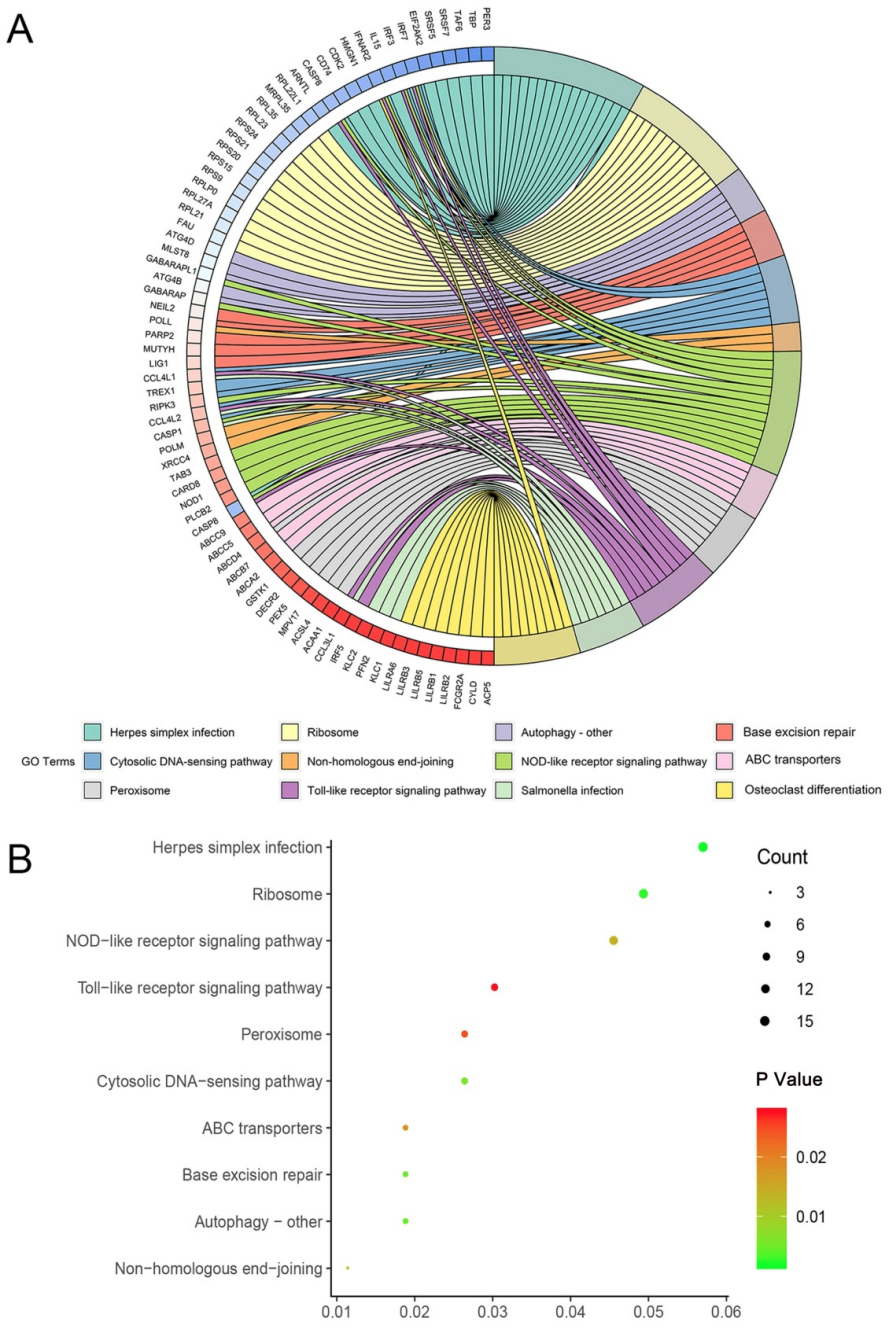
Circle map and dot plot of KEGG enrichment for genes of top 1000 prognosis-related AS events. (A): Circle map. Ribbons with different colors in the right half circle represented top 12 significant KEGG pathways. The 12 top pathways were enriched by genes listed in the left half circle. (B): Dot plot. The color spectrum ranging from green to red indicated an increasing P value and the size of dot represented the number of genes assembled in specific pathway. Each dot in the plot represented specific pathway terms listed in y axis.

Enrichment of genes of top 1000 prognosis-related AS events in KIRC was recorded detailedly in Table 2. Top three significant pathways assembled by these genes were herpes simplex infection, ribosome and autophagy - other (Figure 4).

### Construction of PI models

**Figure 5:**
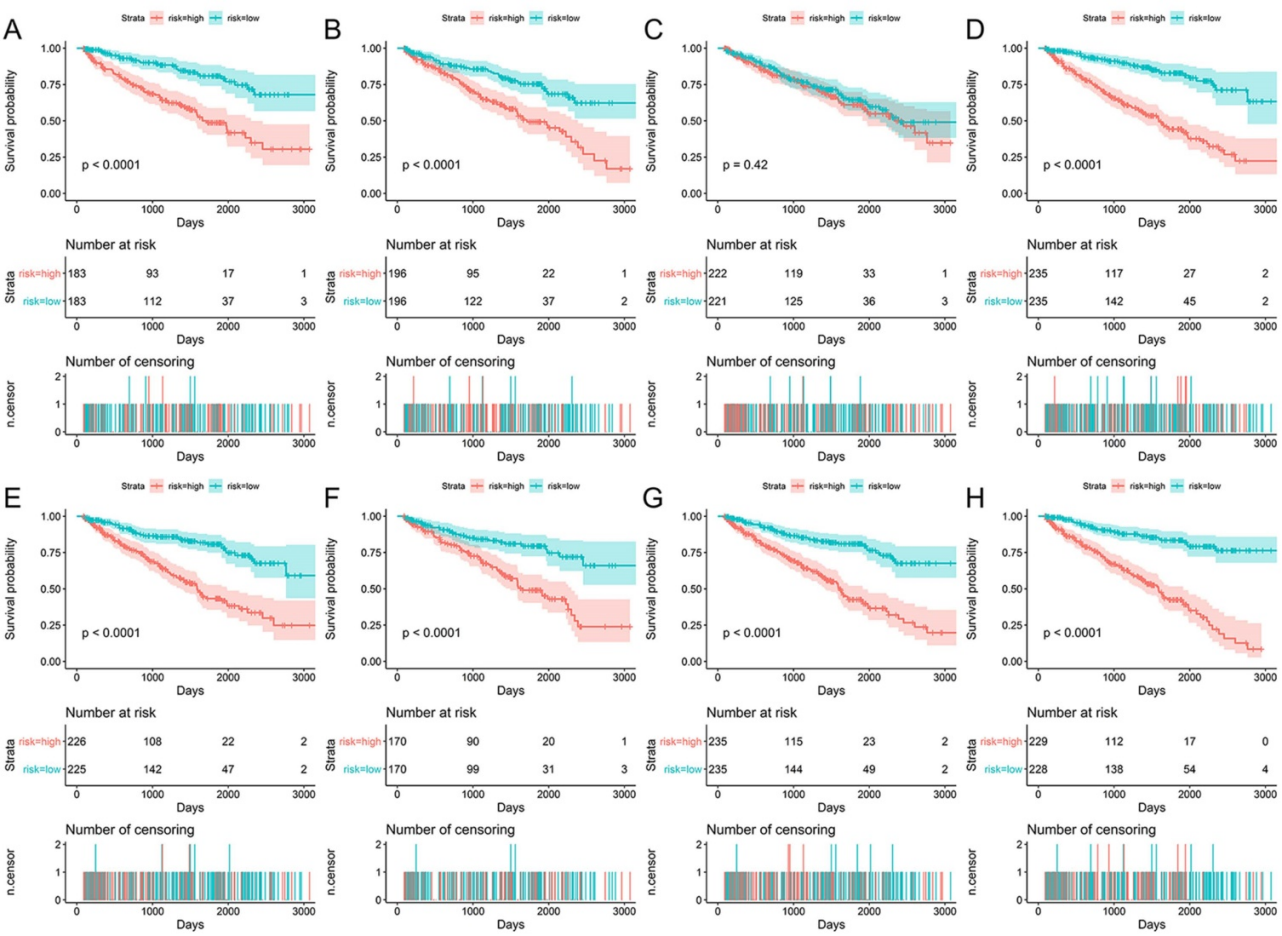
Kaplan-Meier survival analyses for eight prognostic indexes (PIs) in KIRC. Patients in different risk groups of KIRC was divided based on the median value of PSI value of AS events in PI-AA (A), PI-AD (B), PI-AP (C), PI-AT (D), PI-ES (E), PI-ME (F), PI-RI (G) and PI-ALL (H). Numbers of patients in low or high risk groups and censoring data at different time were displayed beneath the Kaplan-Meier survival curves.

**Figure 6:**
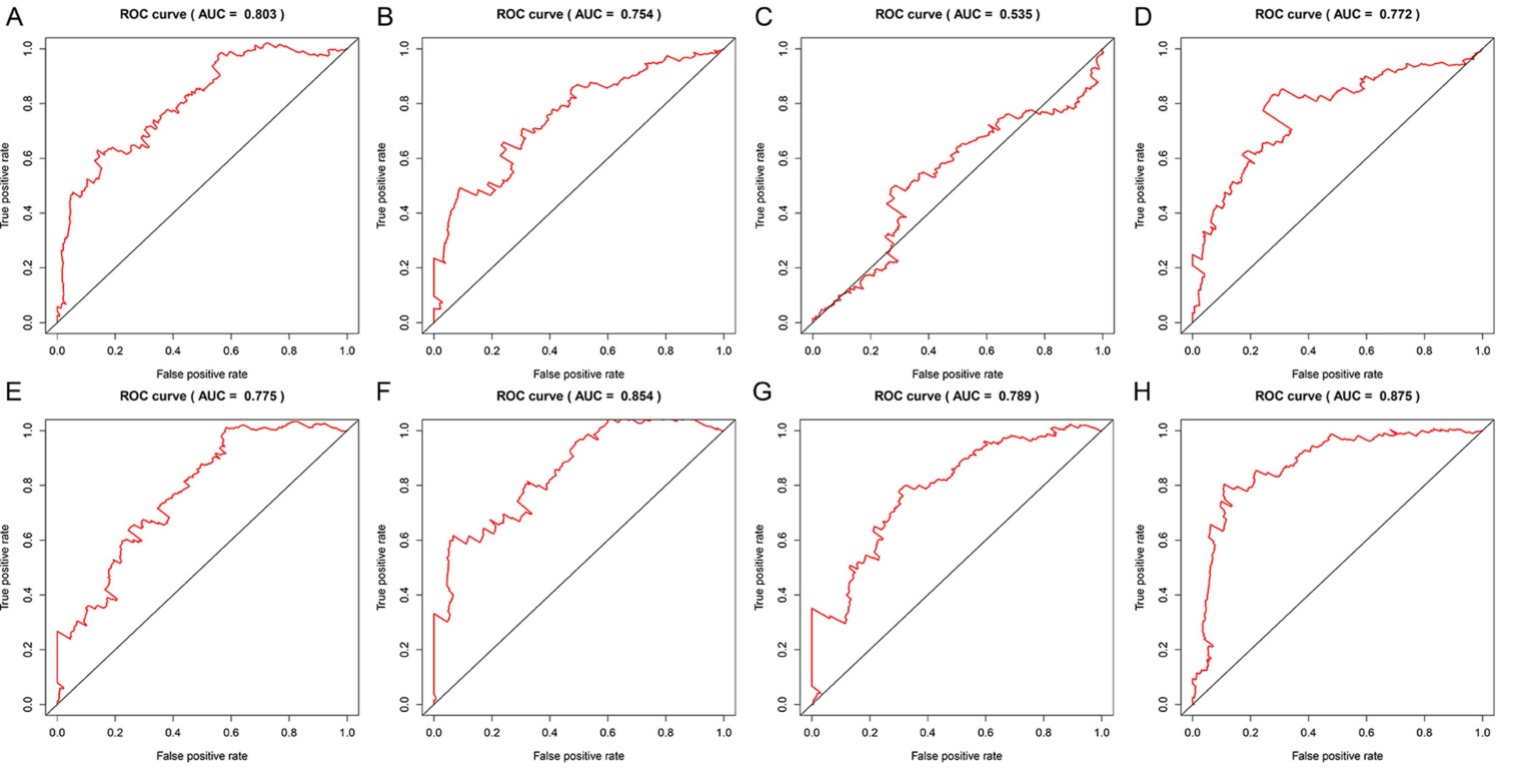
Time-dependent ROC curves for assessing the predicting efficiency of eight PIs in KIRC. A: PI-AA; B: PI-AD; C: PI-AP; D: PI-AT; E: PI-ES; F: PI-ME; G: PI-RI; H: PI-ALL.

Data of AS events from multivariate Cox regression analysis that were adopted as components of PI models were listed in Table 3. Overall, Kaplan-Meier analysis demonstrated the satisfactory risk-stratification ability of PI models in KIRC with the exception of PI-AP (P<0.001) (Figure 5). Time-dependent receiver operator characteristic (tROC) curves further confirmed the predicting efficiency of PI models in KIRC. Emerging as the PI models with strongest predicting efficiency from the moderate property in general level of all PIs, the area under curves (AUC) values of three PIs reached over 0.8 (PI-AA, PI-ME and PI-ALL) and AUC value of PI-ALL was the highest of all (AUC=0.875) (Figure 6).

**Figure 7:**
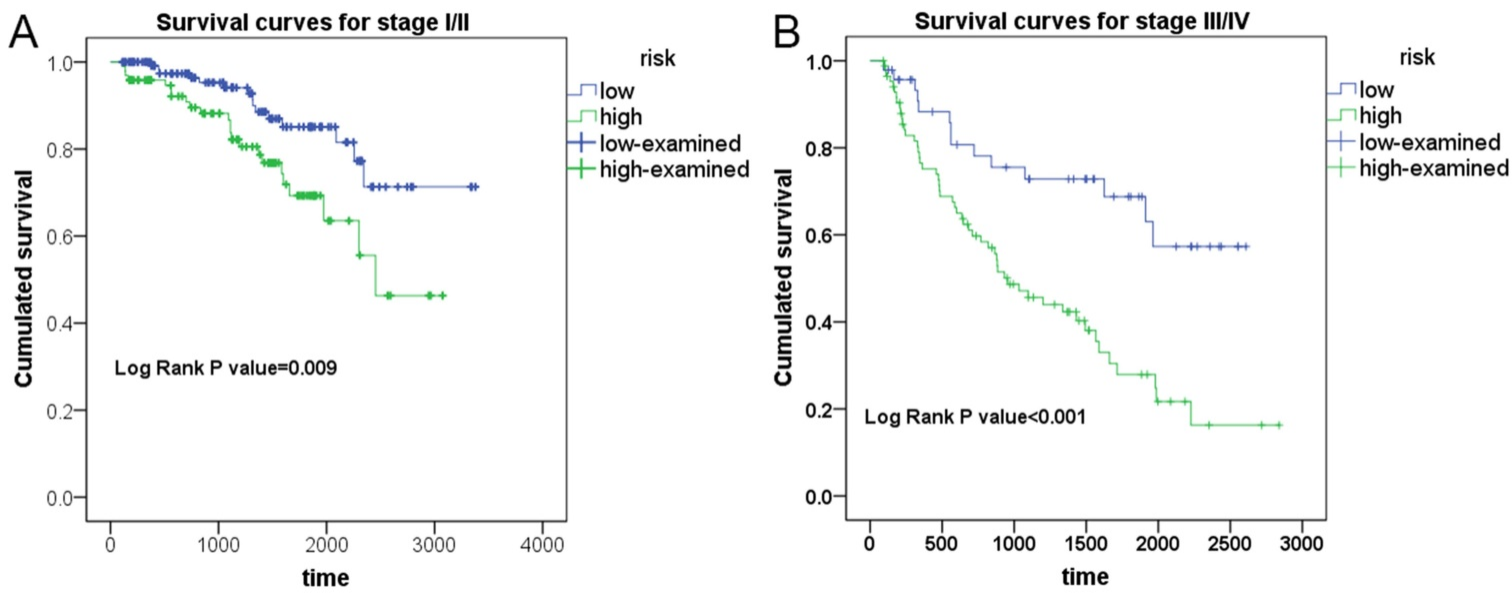
Evaluation of the prognostic value of PI-ALL in early or advanced clinical stage of KIRC patients from Kaplan-Meier survival analysis. A: Differential survival outcome between KIRC patients (stage I-II) in low and high risk groups. B: Differential survival outcome between KIRC patients (stage III-IV) in low and high risk groups

To test the ability of PI models with the strongest predicting efficiency in prognosticating the survival condition of patients in different group of clinical advances, Kaplan-Meier survival analysis was performed to appraise the prognosis-predicting capacity of PI-ALL in stage I-II and stage III-IV groups of KIRC patients, respectively (Figure 7). Expectedly, PI-ALL showed excellent performance in seperating overall survival (OS) conditions of low and high risk RCC patients both in stage I-II and stage III-IV patient groups.

### Regulation network involving splicing factors and prognosis-related AS events

**Figure 8:**
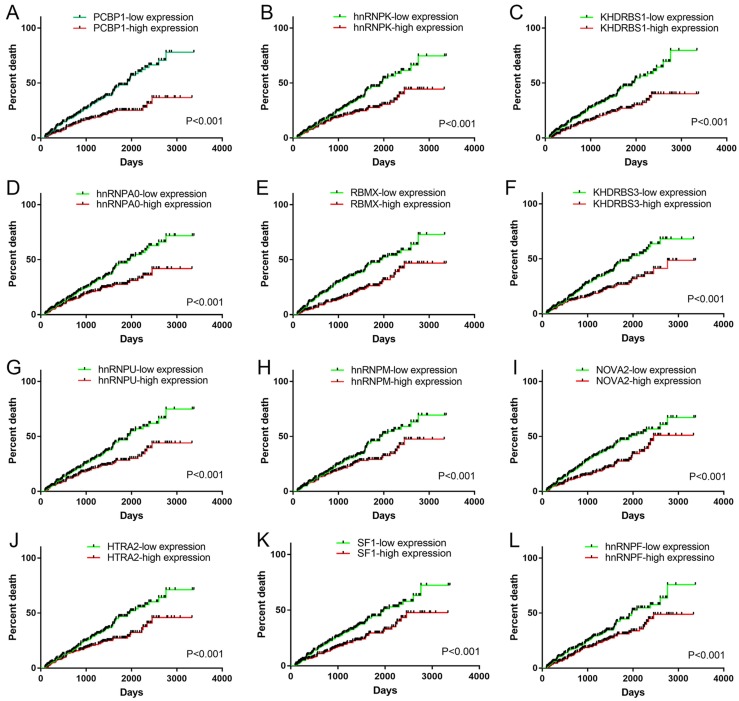
Survival curves of significant prognosis-related splicing factors for KIRC. A: PCBP1; B: hnRNPK; C: KHDRBS1; D: hnRNPA0; E: RBMX; F: KHDRBS3; G: hnRNPU; H: hnRNPM; I: NOVA2; J: HTRA2; K: SF1; L: hnRNPF. Median expression value was set as cutoff value. The survival curves reflected the increase in the percentage of deaths over time.

**Figure 9:**
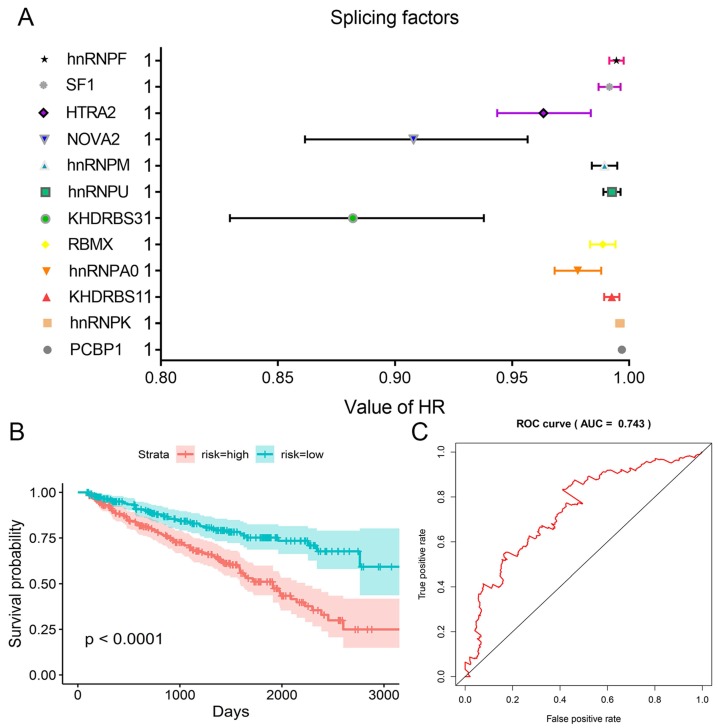
HR values of the 12 significant prognosis-related splicing factors and splicing factor-based PI. A: HRs and corresponding 95%CI were illustrated as nodes and strings for each of the 12 significant prognosis-related splicing factors. B: Kaplan-Meier survival curves for splicing factor-based PI. C: Time-dependent ROC curves for splicing factor-based PI.

**Figure 10:**
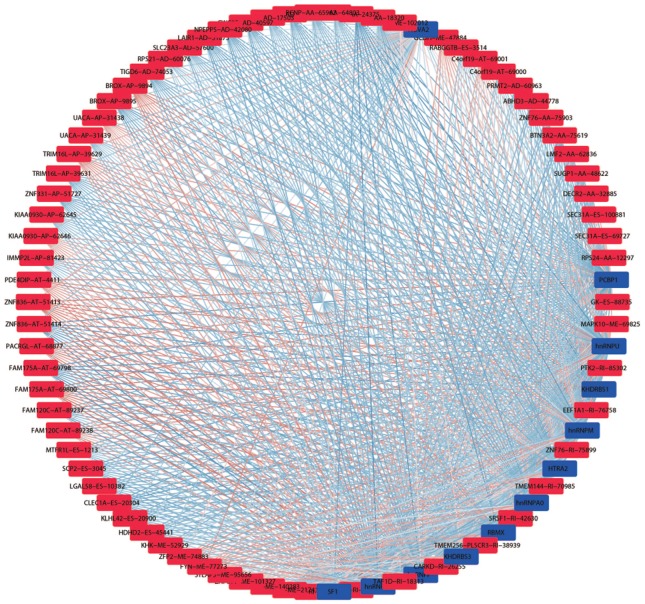
Splicing regulation network in KIRC. Blue and red nots in the network represented prognosis-related splicing factors and AS events, respectively. The gradually changing colors from blue to red indicated transition of correlations between splicing factors and AS events from negative to positive.

We obtained information of 66 splicing factors from SpliceAid2 and univariate Cox regression analysis for all the 66 splicing factors indicated 12 prognosis-related splicing factors in KIRC (Table 4). Interestingly, most of the prognosis-related splicing factors in KIRC heralded relatively good survival outcome of KIRC patients (hazard ratio (HR)<1). A total of 12 splicing factors showed obviously high correlation with the survival of KIRC patients (Figure 8 and 9A). From the 12 significant prognosis-related splicing factors, three splicing factors including PCBP1, KHDRBS3 and HTRA2 were selected from multivariate Cox regression analysis for the construction of splicing factor-based PI and this PI demonstrated moderate prognostic stratification ability (AUC=0.743, P<0.001) (Figure 9B and C). We selected the 12 significant prognosis-related splicing factors (P<0.001) in KIRC to conduct Pearson's correlation analysis of the association between these splicing factors and top ten prognosis-related AS events in KIRC. The correlation network in Figure 10 revealed the positive (red lines) or negative (blue lines) regulatory relationships between splicing factors (blue dots) and AS events (red dots).

### Immunohistochemistry (IHC) of KHDRBS3

**Figure 11:**
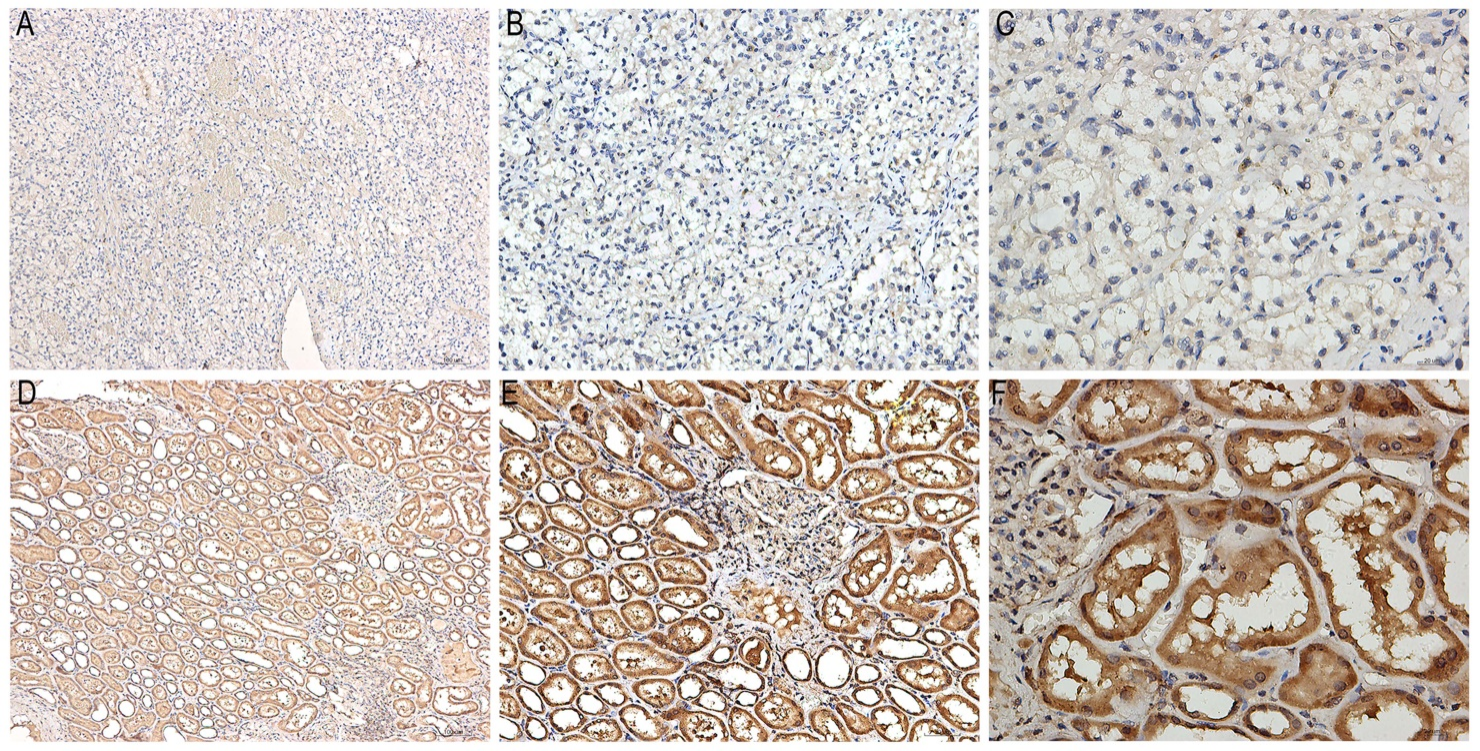
Representative images of immunostaining for KHDRBS3 in KIRC and adjacent normal tissues. A: Immunostaining of KIRC tissues (x100); B: Immunostaining of KIRC tissues (x200); C: Immunostaining of KIRC tissues (x400); D: Immunostaining of normal tissues (x100); E: Immunostaining of normal tissues (x200); F: Immunostaining of normal tissues (x400). The brown area in Figure 11D-F marked the positive staining of KHDRBS3 in adjacent normal tissues.

Immunostaining images of KHDRBS3 in ten pairs of KIRC and paracarcinoma tissues were displayed in Figure 11. KHDRBS3 was primarily expressed in cytoplasm. The positive expression of KHDRBS3 was observed in 0 (0%) and 10 (100%) cases of KIRC and adjacent normal tissues, respectively (Table 5, P<0.0001), which proved that KHDRBS3 was down-regulated in KIRC compared with para-carcinoma tissues.

## Discussion

Currently AS has been proposed as the addition to the growing list of cancer hallmarks 20. Impressed by the profound influences exerted by dysregulated AS events in human cancers, we are propelled to explore the prognostic significance of AS events and the underlying mechanism in RCC. Despite Song J et al. has built a preliminary prognostic model for KIRC based on profiles of AS events; they simply constructed the prognostic model and there was no validation of results or deep exploration of mechanism 21. Moreover, the study of Song J et al. ignored the prognostic role of splicing factors in KIRC and the regulatory relationship between AS and splicing factors in KIRC. Compared to the work of Song J et al., we constructed prediction models based on both prognostic AS events and splicing factors for kidney renal clear cell carcinoma (KIRC). We also constructed regulation network of AS and splicing factors to comprehend the interaction mechanism between AS and splicing factor events on prognosis of KIRC patients. One of the prognostic splicing factor (KHDRBS3) was validated by IHC to be downregulated in KIRC.

Transcriptome-wide analysis of the AS profiling land scape revealed thousands of prognosis-related AS events in KIRC. It is noteworthy that 15 of all the prognosis-related genes possessed four splicing types, involving several genes with critical functions in KIRC such as PLAGL1 and NDRG2. PLAGL1 was originally reported to act as tumor suppressor in several types of human tumors via cell-cycle arresting and pro-apoptotic effect 22, 23. Recently the oncogenic role of PLAL1 in KIRC was discovered that PLAGL1 upregulation in KIRC tissues was positively associated with distant metastasis and poor survival 23. Indeed most of the prognosis-related splicing isoforms of PLAGL1 in the present study indicated worse clinical outcome (HR>1). NDRG2, one of the members of the N-Myc downstream-regulated gene family, was shown by Ma JJ, et al. and Shi W et al. to demonstrate anti-tumor activity in KIRC through inhibiting KIRC cell proliferation and restraining the glycolysis and glutaminolysis process in KIRC 24, 25. Albeit the reported tumor suppressive role of NDRG2 in KIRC, the vast majority of splice variants stemmed from NDRG2 were negatively associated with the OS of KIRC. We conjectured that this phenomena should be attributed to the fact that properties of proteins encoded by different splice isoforms varies from even opposite to each other 20.

To gain deeper insights into the mechanism of prognosis-related AS events in KIRC, we performed KEGG pathway analysis and PPI network for genes corresponding to top 1000 prognosis-related AS events. KEGG pathway annotation revealed key clues for the function of prognosis-related AS events in KIRC that herpes simplex infection, ribosome and autophagy - other were three most significant pathways enriched by prognosis-related AS event in KIRC. Of the thee terms, autophagy, an evolutionary conserved process that refers to the sequestration and delivery of organelles and macro-molecules to lysosome for degradation 26, was closely correlated with human cancers with implications in multiple properties of cancer including motility, invasion, metastasis, epithelial-mesenchymal transition, drug resistance, and immune evasion of tumor cells 27. It is conceivable that the disturbance of above-mentioned pathways caused by AS events were of importance in the pathogenesis of KIRC.

Based on the prognosis-related AS events, we constructed eight prediction models grouped by splicing types. Further evaluation from Kaplan-Meier survival analysis and tROC curves reflected the preferable prognosis-predicting ability of seven PI models (PI-AA, PI-AD, PI-AT, PI-ES, PI-ME, PI-RI and PI-ALL). Although some prediction models composed of miRNAs or mRNAs have been devised by other researchers 28, 29, we are confident of the strong risk-stratification capacity of our PIs. The highest AUC value from the seven PI models was close to 0.9, obviously higher than that of the five-gene signature in the study of Zhan Y et al. 29. Unlike the study of Liang B et al., where the three miRNA constituents for prediction models came from the overlapping parts of OS markers, disease-free survival markers and diagnostic markers 28, AS events included for PI in the present study were selected from two steps: univariate Cox regression analysis and multivariate Cox regression analysis. Methodological differences between our study and the study of Liang B et al. may lead to different AUC values of prediction models. On balance, PIs proposed in our study embodies the novelty of our work in that we are the first group to construct PI models for KIRC from the perspective of AS and PI models in the current work performed well in predicting the survival of KIRC patients.

It is widely acknowledged that splicing factors were key regulators of AS 30. Hence, we further concentrated on prognosis-related splicing factors and their correlation with top 10 prognosis-related AS events in each splicing type. A total of 12 splicing factors were significantly associated with the OS of KIRC, among which splicing factors such as PCBP1, hnRNPK and hnRNPM were engaged in tumorigenesis through guiding alternative splicing programs in various cancers 31-33. One of the splicing factors: KHDRBS3 showed significant decreased expression in KIRC tissues from IHC experiment, Down-regulation of KHDRBS3 was consistent with the indication of good prognosis (HR<1), which indirectly validated the prognostic value of KHDRBS3 in KIRC. The correlation network between splicing factors and prognosis-related AS events depicted the complicated relationships between them. Multiple splicing factors coordinated to mediate prognosis-related AS events, thus affecting the survival of KIRC patients. The creation of correlation network marked a step forward for us in deciphering the regulatory mechanism of AS events in KIRC.

Despite the encouraging findings in the present study, room for improvements remains for this study. The original intention was to conduct survival analysis of AS events in pan-kidney cancers. However, the small number of deaths restrained us from performing survival analysis for AS events in other subtypes of RCC: KICH, KIRP type 1 and KIRP type 2. There is a lack of testing set of for the verifying the prognosis-predicting ability of PIs in KIRC after the construction of PIs. Moreover, the biological function of AS events in KIRC and the relationships between splicing factors and AS events needed experimental validation. All of these defects pointed out directed the future efforts of our work.

In summary, PIs based on prognosis-related AS events were constructed by univariate and multivariate Cox regression analysis of AS profiles in KIRC. Subsequent evaluation results proved that risk-stratification by PI enabled the satisfactory differentiation of survival outcomes in KIRC patients. Additionally, the correlation network between splicing factors and prognosis-related AS events shed lights on the genetic mechanism of KIRC.

## Methods

### Curation and preprocess of TCGA data

AS profiles of RCC patients were generated using SpliceSeq, a java program that provides a clear view of the splicing patterns through accurate alignment of reads and exon-inclusion or skipping splice junction 34. PSI values were calculated by SpliceSeq to quantify seven types of AS events 35: ES, RI, ME, AD, AA, AP, and AT for three major types of RCC (KICH, KIRC and KIRP). We only included AS events that met the criteria of PSI value >75% and standard deviation >0.1 for this study. Clinical-pathological information of RCC was also downloaded from TCGA data portal.

### Survival analysis

Three major types of RCC cases (KICH, KIRC and KIRP) with OS time over 90 days and PSI information of AS events were eligible for the present study; RCC cases were excluded from survival analysis if the criteria listed above was not met. We performed univariate Cox regression analysis R software v.3.5.1 to judge whether the AS events were prognosis-related or not according to the median of PSI value. Then, top ten prognosis-related AS events in each splicing type from KICH, KIRP type1 and KIRP type2 as well as top 50 prognosis-related AS events in each splicing type from KIRC were subjected to multivariate Cox regression analysis in SPSS v.22.0 for the identification of AS events appropriate for the construction of prediction models. Significant prognosis-related AS events from multivariate Cox regression analysis were included as components of PI models corresponding to each splicing type. Specifically, PI-ALL was built based on multivariate Cox regression analysis results of top ten or 50 prognosis-related AS events from all splicing types. The following formula was applied in calculation of PIs: PI=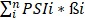 (ß is the regression coefficient). The Kaplan-Meier survival analysis was employed in GraphpadPrism v.7.0 to compare the survival difference of KICH, KIRC and KIRP patients groups divided by the median value of PI. The prognosis-predicting ability of PIs was evaluated by plotting the tROC curves with the aid of survivalROC package in R software v.3.5.1. The predicting efficiency of each PI model was compared at 3000 days for fewer events occurred after 3000 days. Two-tailed P value of less than 0.05 was considered statistically significant. The interactive sets in seven splicing types of AS events were visualized and aggregated by UpSet package of R software v.3.5.1, which exhibited better performance than Venn plot when the number of datasets was more than five 36.

### Interaction network and pathway annotation for genes of prognosis-related AS events

We uploaded genes of top 1000 prognosis-related AS events in all splicing types from univariate Cox regression analysis to online program: STRING v.10.5.0 for the creation of PPI network. The highest confidence (0.9) was assigned to the PPI network in order to guarantee the credibility of results. Genes in the PPI network with the highest connection degrees were defined as hub genes. Kyoto Encyclopedia of Genes and Genomes (KEGG) pathway analysis for genes submitted to STRING was carried out by ClusterProfiler package of R software v.3.5.1. Terms with p value <0.05 were significant pathways enriched by these genes.

### Regulation network involving splicing factors and prognosis-related AS events

Aware of the predominant position of splicing factors in mediating AS, we further investigated the regulatory relationships between splicing factors and AS events for three major types of RCC (KICH, KIRC and KIRP). Firstly, we downloaded information of humankind splicing factors from SpliceAid 2 (www.introni.it/spliceaid.html) 37. Then transcripts per million (TPM) expression values of splicing factors in three major subtypes of RCC were deduced from read count values in TCGA data portal. Subsequent univariate Cox regression analysis was conducted to assess the prognostic value of splicing factors in each major subtype of RCC. Prognosis-related splicing factor were singled out for Pearson's correlation analysis of the association between splicing factors and top ten prognosis-related AS events in each splicing type. Correlation diagrams representing the regulation between splicing factors and prognosis-related AS events for each major subtype of RCC were drawn by Cytoscape v.7.0.

### IHC of KHDRBS3

Ten pairs of KIRC and adjacent normal tissues were collected from the First Affiliated Hospital of Guangxi Medical University during the period of September 2017 to February 2019. The experiments were approved by the Ethical Committee of the First Affiliated Hospital of Guangxi Medical University and written informed consent was signed by each participant. The standard procedures of IHC were described in detailed previously 38. All samples were incubated using rabbit polyclonal anti- KHDRBS3 antibody (1:1000 dilution; catalog no. ab 881155445) overnight at 4°C. Two pathologists unaware of the clinicopathological data assessed the immunostained slides independently. The criteria for evaluation of IHC has been elaborated in previous study 38. Analysis of KHDRBS3 expression patterns in KIRC and paracarcinoma tissues was performed via Wilcoxon signed-rank tests in SPSS v.22.0.

## Figures and Tables

**Table 1:** Clinical characteristics of the 468 KIRC patients.

Variables	Patient characteristics
Age, mean years ± SD	60.18±12.05
Overall survival time, mean days ± SD	1180.71±763.54
Sex, n (%)	
Male	309
Female	159
Prior malignancy	
Yes	68
No	400
Synchronous malignancy	
Yes	3
No	398
Race	
Asian	8
Black or African American	36
White	418
	
Ethnicity, n (%)	
Hispanic or Latino	25
Not Hispanic or Latino	300
Tumor stage, n (%)	
Stage I	232
Stage II	50
Stage III	106
Stage IV	77
Overall status	
Alive	325
Dead	143

**Table 2:** KEGG annotation for genes of top 1000 prognosis-related AS events in KIRC

ID	Description	GeneRatio	Bg Ratio	P value	Q value	Count
hsa05168	Herpes simplex infection	15/259	185/7470	0.001859621	0.31156572	15
hsa03010	Ribosome	13/259	153/7470	0.002447308	0.31156572	13
hsa04136	Autophagy - other	5/259	32/7470	0.004505404	0.31156572	5
hsa03410	Base excision repair	5/259	33/7470	0.005162598	0.31156572	5
hsa04623	Cytosolic DNA-sensing pathway	7/259	63/7470	0.005856499	0.31156572	7
hsa03450	Non-homologous end-joining	3/259	13/7470	0.009102644	0.40355057	3
hsa04621	NOD-like receptor signaling pathway	12/259	168/7470	0.013668241	0.519393161	12
hsa02010	ABC transporters	5/259	44/7470	0.017363984	0.577352452	5
hsa04146	Peroxisome	7/259	83/7470	0.024578315	0.705407061	7
hsa04620	Toll-like receptor signaling pathway	8/259	104/7470	0.027553359	0.705407061	8
hsa05132	Salmonella infection	7/259	86/7470	0.029170969	0.705407061	7
hsa04380	Osteoclast differentiation	9/259	128/7470	0.033416332	0.740728694	9

**Table 3:** Multivariate Cox regression analysis of prognosis-related evets for KIRC

PI model	Splicing type	Gene symbol	AS ID	Exons	From exon	To exon	Hazard ratio	P-value
PI-AA	AA	TAF1D	18320	8.1	7	8.2	1.046	0.005
	AA	GIT2	24375	18.1	17.2	18.2	1.023	0.008
	AA	CEP76	44711	4.1	3.2	4.2	0.972	0.002
	AA	WBSCR27	80035	6.1	5	6.2	1.034	0.003
PI-AD	AD	TTC14	67731	5.2	5.1	5.4	1.016	0.022
	AD	TIGD6	74053	1.2	1.1	2	1.025	0.033
	AD	SLC25A37	83083	2.2	2.1	3.2	1.043	0.003
	AD	TMEM205	47677	2.2:2.3	2.1	2.6	1.029	0.003
PI-AP	AP	UACA	31439	1	null	null	1.017	0.008
	AP	GPR56	36577	2	null	null	0.986	0.041
	AP	KIAA0930	62645	5.1	null	null	1.014	0.006
	AP	HHLA2	66019	2	null	null	1.011	0.009
PI-AT	AT	ZNF814	52354	9	null	null	0.972	<0.01
	AT	AGBL5	52925	16	null	null	0.979	0.004
	AT	EPB41L5	55145	17	null	null	0.984	0.008
	AT	FARP2	58380	18.2	null	null	1.016	0.008
	AT	PCSK5	86633	38	null	null	0.977	<0.001
PI-ES	ES	FAM210A	44743	2	1	3	1.018	0.005
	ES	GK	88735	23	22	24	1.043	<0.001
	ES	RPL13A	264941	2.1:2.2	1	3	1.023	0.008
PI-ME	ME	STEAP3	95656	2|3	1.1	5.3	1.016	0.002
	ME	ZFP2	74883	6|7	3	8	0.971	0.007
	ME	TMEM67	84536	4|5	3.2	6	0.967	0.016
	ME	ZNF415	51689	4|5.1:5.2	1.1	6.1	0.98	0.007
PI-RI	RI	FBXO3	14929	12.2:12.3	12.1	12.4	1.031	0.001
	RI	MTERFD3	24188	1.2	1.1	1.3	1.014	0.021
PI-ALL	AA	RPS24	12297	5.1	4	5.2	0.986	0.044
	AA	TAF1D	18320	8.1	7	8.2	1.024	0.022
	AA	WDR6	64803	4.1	1	4.2	1.02	0.016
	AT	FAM120C	89237	2	null	null	1.019	0.007
	ES	GK	88735	23	22	24	1.023	0.007

Note: AA: alternate acceptor; AD: alternate donor; AP: alternate promoter; AT: alternate terminator; ME: mutually exclusive exons; RI: retained intron.

**Table 4:** Prognosis-related splicing factors from univariate Cox regression analysis

Splicing factor	HR	Lower	Higher	P
PCBP1	0.996925078	0.995801498	0.998049927	8.67E-08
hnRNPK	0.996168459	0.994570885	0.9977686	2.76E-06
KHDRBS1	0.99273841	0.989470394	0.996017219	1.48E-05
hnRNPA0	0.978490809	0.968526893	0.988557231	3.13E-05
RBMX	0.989015616	0.983571555	0.99448981	8.78E-05
KHDRBS3	0.884099178	0.831252165	0.94030595	8.96E-05
hnRNPU	0.992822926	0.989164456	0.996494927	0.000131249
hnRNPM	0.98975287	0.984307009	0.995228862	0.000253324
NOVA2	0.90900193	0.862723176	0.957763199	0.000345376
HTRA2	0.963833853	0.944050899	0.984031367	0.000499006
SF1	0.991819702	0.987122205	0.996539553	0.00069623
hnRNPF	0.994732543	0.991670876	0.997803662	0.00078522

HR: hazard ratio

**Table 5:** KHDRBS3 expression in KIRC and paracarcioma tissues

	KHDRBS3 expression		P-value
	negative, n (%)	positive, n (%)	
KIRC tissue (n=10)	10 (100)	0 (0)	<0.001
Paracarcinoma tissue (n=10)	0 (0)	10 (100)	

Note: KIRC: kidney renal clear cell carcinoma.

## Abbreviations

RCC: renal cell carcinoma
AS: alternative splicing
KICH: kidney chromophobe renal cell carcinoma
KIRP: kidney papillary renal cell carcinoma
KIRC: kidney clear renal cell carcinoma
TCGA: the cancer genome atlas
AA: alternate acceptor site
AD: alternate donor site
AP: alternate promoter
AT: alternate terminator
ES: exon skip
ME: mutually exclusive exons
RI: retained intron
PSI: percent spliced in
OS: overall survival
PI: prognostic index
tROC: time-dependent receiver operating curves
AUC: area under curves
HR: hazard ratio
PPI: protein-to-protein
KEGG: kyoto encyclopedia of genes and genomes (KEGG)
